# Hong Kong veterinarians’ encounters with client-related stress – a qualitative study

**DOI:** 10.3389/fvets.2023.1186715

**Published:** 2023-11-30

**Authors:** Camille K. Y. Chan, Paul W. C. Wong

**Affiliations:** Department of Social Work and Social Administration, The University of Hong Kong, Pokfulam, Hong Kong SAR, China

**Keywords:** mental health, veterinarians, client-related stress, occupational stress, communication, expectation, cyberbullying

## Abstract

**Aims:**

Limited research has explored the stressors experienced by veterinarians in East Asia. Metropolitan cities like Hong Kong may have overlooked factors that significantly contribute to veterinarians’ stress. This paper examines how client-related stressors and contextual factors contribute to the stress levels of Hong Kong-based veterinarians.

**Methods:**

Veterinarians from small domestic animal practices in Hong Kong were recruited using purposive and targeted snowball samplings until thematic saturation was achieved. Semi-structured in-depth interviews were conducted, audio-recorded, transcribed, coded, and analysed using reflexive thematic analysis with an interpretivist paradigm based on Heidegger’s phenomenological approach.

**Results:**

A total of 18 participating veterinarians described the expectations of and communication with the animal-health enthusiast (AHE) community, including clients, pet owners, and animal lovers, as their primary source of stress. Discrepant expectations and miscommunication between the AHE and veterinary communities, along with contextual factors, such as convenience in clinic switching, negative online reviews, and the relatively short history of the veterinary profession in the multilingual city, were identified as contributing factors to their stress. Recent socio-political events and the pandemic further intensified societal emotions, leading some clients to project frustrations onto perceived authority figures, including health professionals.

**Conclusion:**

The study highlights that client-related stress extends beyond the health of animal patients, encompassing the expectations of the AHE community, which requires professional-level communication skills to build a mutually respectful three-party relationship. Besides, Hong Kong’s unique contextual and historical factors subtly but chronically impact veterinary practices, which can be easily overlooked. Recognising these influences and how they interact is crucial for collaboration, within and beyond the profession, at the policy level to improve veterinary training and practices.

**Implications:**

Our findings highlight the importance of expectation management and improving communication quality to foster healthy relationships among animals, the AHE community, and veterinary professionals. These efforts are believed to alleviate veterinarians’ stress and enhance the well-being of all parties involved. We recommend incorporating effective communication and self-care discussions into the veterinary curriculum and advocating ongoing training for practising veterinarians. At the community level, encouraging open dialogues between animal caregivers and veterinary professionals can help address expectation discrepancies.

## 1 Introduction

Veterinarians face unique challenges as they provide healthcare for their animal patients, who cannot communicate in human language. Common stressors such as long working hours, unpredictable workload, and direct engagement in euthanasia (1–5) contribute to the poor mental health of many veterinarians. Additionally, the delicate balance required to meet clients’ needs while operating within medical limitations (6, 7) further adds to their stress. Meeting unrealistic client expectations, particularly when clients question the cost of treatment and the expertise of the clinicians (8), appears to be a significant stressor facing veterinary professionals. On the other hand, clients place great importance on veterinarians’ ability to understand their concerns, to provide personalised information, and to guide their decision-making about their animals while respecting their clients’ autonomy (9). Despite the recommendation to integrate partnership-centred care in their daily practice (9, 10), veterinarians worry that the surge in cyberbullying, involving intentional aggression towards veterinarians and its publicisation, may further strain the veterinarian–client relationship (11–13). The complex interplays inherent in veterinarians’ duties can significantly impact their mental well-being and therefore warrant further investigation.

Hong Kong, being one of the most densely populated cities in the world, with high rental costs, triggers intense business competition among the 200 veterinary clinics (14), leading to stress among veterinarians (1). There are approximately 1,100 registered veterinarians in Hong Kong (15), with more than 90% providing services for cats and/or dogs (8). While most Hong Kong pet owners are satisfied with the quality of veterinary medical service (8), some have high expectations, such as immediate improvement of their sick animals (1), which can be stressful for veterinarians. Despite the profession acknowledging that a quality veterinarian–client–patient relationship is critical to providing optimal care to patients and clients (10, 16–18), the veterinary curriculum has been criticised for its lack of the essential humanistic aspects necessary for delivering empathic communication (1).

Complaints against veterinarians to the Veterinary Surgeon Board of Hong Kong (VSBHK), an independent statutory body, are predominantly related to professionalism and communication issues (19). Resolution of these cases can take several years, depending on the complexity of the case (19). Although clients may turn to unofficial channels on the Internet to voice their grievances due to frustration with delayed official responses, veterinarians express concern about the unpredictable magnitude of these one-sided narratives, which may mislead readers (11). Furthermore, veterinarians are bound by codes of conduct that prohibit them from making public statements commenting on specific cases, rendering them defenceless and adding to their stress levels (1).

The demanding nature of the veterinary profession can have detrimental effects on their mental health. Compared to other professionals, veterinarians are at heightened risk of suicidal behaviour due to their accessibility to lethal drugs and their knowledge of how to use them (3, 20, 21). However, most stress-related research on veterinarians has been conducted in Western countries, leaving a significant gap in knowledge regarding conditions in Asia (1). Given the unique contextual factors present in Asian cities like Hong Kong, little is known about how a competitive business culture and negative online reviews may add to veterinarians’ stress and impact their mental well-being. This paper aims to explore how the cultural influences in Hong Kong contribute to client-related stress among veterinarians through qualitative research.

## 2 Materials and methods

This paper is part of a mixed-methods study. The overall study includes a qualitative exploration aimed at identifying stressors that impact the well-being of Hong Kong veterinarians, followed by a quantitative survey to investigate the prevalence of mental ill-health among them. This paper reports parts of the findings of the qualitative component of the overall study, which solely focuses on client-related stressors. Findings relevant to intra-profession stressors of this qualitative study were reported elsewhere (1) and how the participated veterinarians cope with the stressors will be reported elsewhere ((22) in progress).

This paper is reported according to the Consolidated Criteria for Reporting Qualitative Research reporting guidelines (23). The study procedure was approved by the Human Research Ethics Committee of the University of Hong Kong (reference number: EA200192).

### 2.1 Philosophical underpinning

Our methodology follows Heidegger’s phenomenological approach, which centres on the interpretation and contextual significance of human experiences (24). Within the framework of modern capitalist society (25), we acknowledged the coexistence of multiple social realities (26, 27) and actively facilitated a process of knowledge co-construction between the researchers and participants. This collaborative approach allows us to collectively make sense of the shared experiences of veterinarians (26).

### 2.2 Recruitment and sampling

Veterinarians registered in VSBHK whose primary patients are small domestic animals and who work in client-facing roles were invited to participate in this study. Those who had retired or were not practising in Hong Kong at the time of recruitment were excluded.

E-invitations were disseminated via social media and personal networks and with the assistance of the Hong Kong Veterinary Association. Potential participants were asked to follow an online link or a QR code to a survey platform (28), where they were e-screened. Eligible participants were given the research information sheet, asked about their background, and gave their consent to partake in the research. The background questions included gender, ethnicity, country of graduation, years of practice, broad-certified specialist status, Cantonese language proficiency, type of practice and employment, and contact information.

Due to Hong Kong’s bilingual environment, where English is typically introduced to the citizens at an early age and good command of the language is prevalent, especially among professionals, and given that all veterinarians received their education primarily in English-speaking countries abroad, all research materials, such as the invitation, information sheet, screening questions, and consent form, were solely presented in English and not Traditional Chinese.

We adopted purposive sampling and targeted snowball sampling methods to achieve maximum variation among participants. This involved sending out postal invitations to potential participants and accepting referrals from participants in the latter stage of recruitment. Specifically, we sought referrals targeting specific background characteristics that were lacking among our recruited participants, such as veterinarians who were under disciplinary investigation or were outspoken on social media, to ensure adequate diversity among our participants. Based on our ongoing data analysis, we determined the closure of recruitment once thematic saturation was reached.

### 2.3 Interview guide and interviewer’s characteristics

In preparation for the semi-structured in-depth interviews, an interview guide was developed by drawing insights from published literature (8, 29–33), desk research on social media platforms (11, 34), and informal discussions with connections within the veterinary profession. The interviews focused on topics such as the dynamics of the veterinarian–client–patient relationship, stressors and rewards of being a veterinarian, and coping strategies for their daily stress. The interviews were conducted using a bilingual guide in English and Cantonese and started with the question, “What made you become a veterinarian?” with prompts that asked about their experiences and the challenges they faced through a series of “Why?” and “What makes you say that…?” inquiries. To gain insight into client-related stress and the influence of social media, a case vignette was presented involving a dissatisfied client leaving negative comments about a veterinarian. Prior to moderating all interviews, the first author conducted a pilot study with an experienced veterinary nurse, who was believed to have a more neutral stance on the studied topic, to assess the interviewing procedure and the relevance of the interview guide. The interview guide is included as a Supplementary file.

All interviews were conducted by the first author, who was a doctoral candidate at the time of the interviews. The interviewer, who is biologically female and proficient in Cantonese (native language) and English (completed tertiary education in Australia), also possesses more than 8 years of experience interviewing medical professionals in the market research industry and is a provider of human emergency medical services. The two authors did not have a veterinary background, and it was believed that their outsider stance could facilitate the clarification of the meanings underlying the interviewees’ actions and thoughts.

### 2.4 Data collection

The interviews took place in participants’ clinics, a university classroom, or a coffee shop at mutually convenient times between December 2020 and April 2021, when public places were less crowded during the pandemic. Prior to the interviews, participants provided written and verbal consent and were informed that the interviews would be audio-recorded and they had the right to decline answering questions if they were uncomfortable, cease the interview at any time, or withdraw from the study. Field notes were taken during each interview.

The interviewer practised reflexivity by keeping a reflective journal, written in English, to recognise any unintentional power dynamics before and after the interviews and during data analysis (35). The audio-recorded interviews were transcribed by the interviewer in the languages in which they were conducted, i.e., English and/or Cantonese. Participants were invited to review the transcripts to ensure the accuracy and validity of the content. The second author then listened to all audio recordings of the interviews and discussed with the first author throughout the data analysis and reporting processes.

### 2.5 Data analysis

The collected data were analysed from an interpretivism paradigm, focusing on understanding and interpreting social phenomena through individual experiences and subjective meanings (36). Data analysis was an iterative process, which ensured the extensivity of inductive and deductive themes until thematic saturation was reached (37). This study utilised the six stages of reflexive thematic analysis (38, 39), involving (i) familiarisation with the datasets, (ii) coding, (iii) generating initial themes, (iv) developing and reviewing themes, (v) refining, defining, and naming themes, and (vi) writing up the findings. The coding and data analysis process was discussed between the first and second authors, with an independent reviewer, who was also a doctoral candidate, assisting with checking the codes to ensure they accurately reflected the transcripts’ contents and reviewing stage 3 of the analysis. This process was performed in the English language.

In the first stage, the first author familiarised themselves with the data by conducting and transcribing all interviews, followed by reviewing the audio recordings, transcripts, and interview notes collectively. A two-stage coding process was implemented in stage two of reflexive thematic analysis. The initial phase involved reading each transcript line-by-line (40) to examine both its content and meanings, where 326 codes and 3,413 data units were identified. The researcher then compared these codes across participants and revised them for consistency, resulting in 313 codes and 3,032 data units. Initial themes were generated by connecting codes that shared similar ideas and meanings across different contexts, providing broader perspectives beyond the codes. This stage generated 10 themes and 44 sub-themes. In stage four, developing and reviewing themes involved the researcher reassessing the relevance of each theme to the codes, comparing them inductively within the dataset and deductively with the existing frameworks and literature. This stage led to the establishment of a conceptual model called the Ecology of Veterinarians’ Mental Health Model. This model highlighted two stakeholder groups, namely the veterinary community and animal-health enthusiast (AHE) community, connected by two overarching concepts—communication and expectations—which were presented in each theme. Before writing up the research findings, the researcher refined the principles, scope, boundaries, uniqueness, and contribution of each theme to the overall analysis. This stage ensured the themes were informative and concise and had catchy names that clearly defined their core concept. The overall qualitative analysis generated six themes and 24 sub-themes. Among the six themes, one theme was related to intra-professional stressors, one theme was about the participants’ coping behaviours, and four were related to AHEs and clients, as reported in Table 1.

**Table 1:** Four client-related themes and sub-themes on Hong Kong veterinarians’ stress and mental well-being between December 2020 and April 2021.

Themes	Sub-themes
Different roles, same love	Navigating human bonds; Seeing through clients’ eyes; Not just the good medicine; Trust unleashed.
Veterinarians: guardians of animal health	You are the vet!; The newbie or the knowbie?; The healing hands.
Beyond control	Culture’s toll; Get down to business; Unpunished accusers; Mental health? Who cares?; The macro-bizarre;
Veterinarian’s heart: feeling it all	Hear me cry; Feeling blue; The show must go on.

Quotations cited in this article were translated into English during the manuscript preparation by the first author and proofread by the second author. Redundant words were removed to improve the clarity of the extracts with no intention of changing their meanings. Since data analysis occurred concurrently with participant recruitment, preliminary findings were shared with two respondents following their interviews to ensure the relevance and legitimacy of the findings.

## 3 Results

### 3.1 Participants

Out of the 18 veterinarians who showed interest in the online invitation link, 7 did not provide their contact details. In total, 11 were contacted and agreed to participate in the research. Additionally, 5 more participants were recruited through referrals, and 2 were invited via mail invitation. No eligible participants withdrew from the study and only 1 participant declined to respond to one question during the interview. The interviews lasted an average of 1 h and 40 min, ranging from 55 min to 2 h and 8 min.

A total of 6 men and 12 women were interviewed between December 2020 and April 2021. Among them, 10 identified ethnically as Asians, 6 as Caucasians, and 2 as being from a different ethnic background. Most participants reported that they obtained their earliest veterinary qualifications in Australia (*n* = 8), the United Kingdom (UK) (*n* = 5), or the United States of America (US) or Canada (*n* = 2), and all had been practising for more than 2 years. Most participants worked as veterinarians without a speciality (*n* = 16), 7 were practice owners or held senior management positions, and 11 participants were employees in their practices. The participants predominantly worked in practices solely owned by veterinarian(s) (*n* = 12), with 4 working in practices co-owned by non-veterinarian business partners and 1 working in a practice solely owned by non-veterinarian business owner, and 1 participant worked in a non-government organisation that provides veterinary services for pet owners and stray rescued animals.

### 3.2 Overview

As mentioned previously, six themes were generated from the overall qualitative component of the mixed-methods study. This paper only reports the themes that are client-related. Specifically, our analysis revealed that veterinarians’ stress extended beyond individual clients to the AHE community, which includes pet owners and animal lovers who may or may not own a pet, as described in theme one. Participants also reported facing distinct stress in meeting the expectations of this community and the challenges of effective communication, as highlighted in theme two. Furthermore, our findings in theme three suggest that contextual factors, such as the competitive business environment, political climate, and cyberbullying, contribute to participants’ overall stress levels. Moreover, participants highlighted that stress arises from unfulfilled expectations and communication breakdown, as showcased in theme four. We will discuss these findings in-depth and explore the implications of client-related stress on veterinarians in the following sections.

### 3.3 Theme 1. Different roles, same love

All participants recognised that clients, pet owners, and animal lovers all played a role in elevating their stress levels. Although helping animals is a veterinarian’s primary responsibility, one participant suggested that *“animal care is the easiest part! Handling aggressive animals, although, may require sedation, but what’s stressful is that many clients dislike the idea of sedation and the fact that I’ll probably upset them”* (Participant 07). While the roles of clients, pet owners, and animal lovers are not mutually exclusive, respondents believed that each group had certain expectations from veterinary professionals, which contributed to their stress and adversely affected their well-being.

Some participants underscored the additional challenges when serving a client who is not the animal’s owner. One participant noted that *“it is quite common in Hong Kong that an animal is brought in by individuals who do not live with them… we rely a lot on looking at the animal and on what the owner reports to us”* (Participant 02) and criticised clients who are unfamiliar with the animal patient and communicate poorly with the animal’s caretakers or owners as barriers to providing quality care, resulting in the pet owners’ dissatisfaction.

The presence of patients with multiple owners poses comparable hurdles for our participants as they strive to meet the expectations of each owner. A few participants were concerned that poor communication among owners could jeopardise treatment direction, thereby negatively impacting clients’ medical services experiences. A participant recalled an instance where they would ask their clients if euthanasia is known and agreed upon by all family members and suggested that *“it’s incorrect for some people to say ‘I do not want to tell the kids’ because they should ensure that everyone was aware of the animal’s condition and the decision being made”* (Participant 06).

“Let us say the animal is owned by the daughter and the dad is paying [for the medical expenses]. The daughter is okay because she knows you have tried everything… But the dad at home is furious… all he knew was that he had to pay a lot of money and his daughter is super upset. They would want to blame somebody [us] for it’” (Participant 18).

It is noteworthy that our participants also reported that animal lovers who do not own an animal have an important role in veterinarians’ stress due to their accessibility and participation on social media platforms. Many participants believed that these bystanders may have mistaken their comments for personal expressions without realising the damage they caused. One participant highlighted that *“it’s not just an opinion when your full name is posted. Bystanders on a forum encourage them [the author of the post] to file a lawsuit based on what’s being said in these negative comments [without knowing our side of the story]”* (Participant 08). All participants voiced their concerns about cyberbullying as they worried its borderless and timeless nature could damage the profession’s reputation. A participant recalled that *“that client kept talking about what she thinks happened and it went on for about 3 years. It [what she has said] was far from the truth”* (Participant 03).

“Have you seen any of the 24 h veterinary clinics with good reviews? Well, there are none! We are all ‘black shops’ and ‘scams’… However, having to go to emergency principally means that [the animal is suffering from] something that is life-threatening. The animal could die. They seemed to have forgotten our side of the story” (Participant 14).

Our research revealed various stressors faced by our participants in their interactions within the AHE community. Beyond the challenges involved in providing medical services to animals, veterinarians must also navigate the complexities of managing the human aspects of veterinary medicine. Meeting diverse expectations and communication challenges within this community can hinder the delivery of quality care and result in client dissatisfaction. Additionally, participants expressed worries about the impact of social media, as cyberbullying and negative comments can harm veterinarians’ reputations and provoke protracted disputes based on misinformation. Some participants stressed the importance of informed decision-making and agreement among all owners involved in an animal’s care.

### 3.4 Theme 2. Veterinarians: guardians of animal health

This theme revealed that both expectations and communication emerged as significant client-related stressors among the veterinarians we interviewed. In the following sub-sections, we elaborate on the findings related to expectations and communication separately, providing a comprehensive understanding of how these factors contribute to the stress experienced by veterinarians in Hong Kong.

#### 3.4.1 Managing self and others’ expectations

Many participants believed that the AHE community expects veterinarians to be all-rounders, surpassing the expectations they have for human medicine clinicians. Participants illustrated this situation with a scenario: *“If someone visited their GPs for an ear problem that did not improve, they’d probably see a specialist after… but when it comes to their pets, instead of going to a specialist, they expect us to handle everything and be good at everything”* (Participant 12). All participants criticised that their efforts went unrecognised by some members of the AHE community, with one participant claiming that *“clients tend to give us attitude and take our efforts for granted”* (Participant 04), leading them to question the worthiness of staying in the profession.

Several participants believed that some members of the AHE community lack comprehension regarding the medical constraints of the veterinary profession. Nearly all participants referred to comments like *“clients seem to regard their pets as objects, and vets are here to fix what’s broken”* (Participant 05), regardless of patient prognosis. Many participants also considered the strength of the human–animal bond a significant factor that affects clients’ expectations of veterinarians, as one explained that *“clients’ love for animals can be as strong as their love for a child… there’s a lot of care and compassion. But expectation and severe love can turn very rapidly to anger and disappointment, and obviously, complaints”* (Participant 09).

“To them [clients], taking their pets to a vet is equivalent to repairing a car. If your pet is not fixed, it means your vet does not know what they are doing, or they are trying to scam you, or they have screwed up. But that’s not how medicine works. While we try our best to diagnose and treat, I can never guarantee the results” (Participant 18).

Our study has not established a consensus on participants’ perceptions of whether clients’ economic wealth and educational level are linked to their expectations of the profession. Some participants suggested that *“dual-income-no-kids clients are often well-educated and happy to work with our advice”* (Participant 17), while others contend that highly educated professionals, such as lawyers, doctors, and nurses, may exhibit a sense of entitlement towards expeditious attention and services. However, all participants admitted that veterinary medical costs can be unaffordable to some and that clients’ willingness-to-pay is not dependent on their affordability but on the bond they have with the animal.

Clients’ unrealistic expectations emerged as a significant stressor for veterinarians in our study. Participants highlighted that the AHE community often expects veterinarians to have expertise in all areas, yet their limited understanding of the veterinary profession oversimplifies their role, viewing them as mere “fixers” regardless of the animal’s prognosis and medical constraints. They further noted that added stress can arise from managing patients experiencing treatment delays and clients with financial constraints. Furthermore, participants noted that the strong human–animal bond within the AHE community intensifies emotions when unexpected medical outcomes occur, leading to anger, disappointment, and a breakdown of trust between veterinarians and the community. This breakdown of trust can manifest as disrespectful behaviours that can negatively affect the stress levels experienced by the profession.

#### 3.4.2 Effective communication

Many participants indicated that communication is the most important skill needed in veterinary medicine, which they were unaware of prior to joining the veterinary workforce. Some observed that *“young vets cannot understand the need to balance medical aspects and consideration with clients’ requests”* (Participant 03), and that it takes time and experience to effectively engage with clients. One participant highlighted that *“the curriculum only prepares students scientifically but not in how they deliver that information to clients… the emphasis was all always on the theory of being a vet and not the practice of being a vet”* (Participant 06) and a few participants worried some of their peers’ confidence could come off as egotistical and arrogant.

“I think the trust has to do with the rapport that’s built between you and the client. There could be rapport within the first 10 min [of the consultation]… but then it’s very difficult for anybody to build rapport with a client when you are young and not confident” (Participant 09).

Non-Cantonese-speaking participants believe they are more prone to miscommunications. One participant suggested that *“building a bond with an English-speaking owner is a lot easier because I’m not being translated”* (Participant 09). On the other hand, a few participants considered such language barriers as a protective means against clients’ burst of emotions because *“if clients are angry, they will be angry in Cantonese… they are going to be angry with the translator, and that’s easier for me to take a step back from”* (Participant 15).

While most participants claimed they would persuade their clients to make medical decisions that best serve animal welfare, they also believe that their short consultation time has a role in the lack of clarity in communication. One participant explained that *“some cases will require more than the 15 min we have… So, if you have an emergency case, you’ll get scold at if you did not check them in, and your next client will scold you if you do”* (Participant 07). Participants suggested that respecting clients’ decisions is also important and stated that *“once we have discussed the options and they have made a decision, I think my job is to reassure them they have made the right ones”* (Participant 15), and through this, they can build a loyal customer base.

Our participants recognised that effective communication is paramount in veterinary medicine for establishing trust and strengthening client relationships. However, they also noted challenges in balancing medical aspects of their animal patients with clients’ requests, which their veterinary education may not adequately address. This especially impacts young veterinarians due to their lack of confidence and experience. Language barriers posed another stressor for non-Cantonese-speaking participants, noting difficulties in navigating communications and building rapport through a translator. Additionally, some participants suggested a busy consultation schedule can hinder effective communication and may compromise the quality of veterinary medical care. Despite these obstacles, participants emphasised the importance of empathy, encouraging informed decisions, and respecting clients’ choices as key factors in establishing trust and fostering client loyalty.

### 3.5 Theme 3. Beyond control

Our findings indicated that the interplay of contextual and socio-political factors in Hong Kong can induce stress among our participants. A few participants put forward the notion that the AHE community’s knowledge of the veterinary profession is limited due to Hong Kong’s short history of veterinary medicine, which may have led to elevated stress levels among veterinarians. One participant remarked, *“Generations of Europeans have had dogs and interacted with vets… But there was no vet school in Hong Kong until recently. Clients’ assumptions about how vets work is probably relatively new here”* (Participant 18) and that *“animals can come in an absolutely horrible condition. Some of this is deliberate cruelty, but some of it is just ignorance… or when people not realising how ill and how painful the animal is”* (Participant 12). Furthermore, a few participants believed Hong Kong’s decent public healthcare system may account for clients’ misaligned expectations of veterinary medical costs, as one highlighted that *“[veterinary service] is high-quality medical care that is not subsidised by the government”* (Participant 09).

“In Hong Kong, we are fortunate to have a very good healthcare system, which pays for the majority of people for most things. Veterinary services come at a cost, and that cost is time. So, a human can get their knee surgery done in 5 months, but for a puppy, we can schedule their knee surgery tomorrow or next week” (Participant 10).

Participants found that Hong Kong clients are less confrontational than those in Western countries. They suggested that *“the confrontational aspect of the Western culture is more like ‘no, you cannot do this’, whereas Hong Kong clients are more like ‘sure, that’s fine’ and then talk behind your back”* (Participant 07). They reasoned that *“[during face-to-face confrontation] one has to be braver to be out there shouting at the vet or the receptionist”* (Participant 12) and suspected its possible linkage with the increasingly common cyberbullying phenomenon. Some participants observed traits of patriarchy and white supremacy in the AHE community, where *“clients are nicer to white male vets and nurses… But there’s also a possibility that they do not speak fluent English to outsmart them”* (Participant 13). However, one participant also noted that *“there is some level of positive racism towards white people in Hong Kong, but it is also much easier to blame a foreigner when things go wrong, because there is no direct communication”* (Participant 18).

Some participants observed that their job title may also play a role in clients’ behaviour. One participant explained that *“a client could be abusive towards nursing staff, and the minute they walk into my consultation room and see me, they are sweet as honey”* (Participant 01). Moreover, nearly all participants brought up age bias in the veterinary profession. One participant highlighted that *“vets who look young are definitely underprivileged”* (Participant 05) as clients tend to have less trust in younger-looking faces, and that *“the community assumes older veterinarians are more knowledgeable, which is common but not necessarily true”* (Participant 12).

In our findings, all participants believed that clients’ perceptions of what is best for their pets may differ from those perceived by veterinarians. Many suggested that, for instance, it is common to find clients who are reluctant to euthanasia, even if their animal has a poor prognosis, while some participants suspected that Hong Kong’s cultural and religious beliefs may have compounded patients’ unnecessary suffering. One participant illustrated frustration over the fact that *“if you have an animal that’s suffering and dying a slow death over a few days and the owners refused to put it to sleep, you are actually being cruel. You’re doing everything as a vet that you should not”* (Participant 12). On the other hand, a few participants claimed to feel the need to constantly remind themselves not to pass judgment on their clients’ decisions because they may have faced difficulties that their attending veterinarian is unaware of.

Another common observation among our participants is clients’ vet-hopping behaviour. All participants observed that *“clients would not stick with the same vet”* (Participant 08) and claimed that *“clients will try out different clinics with good online reviews. They frequently seek for second, third, or fourth opinions”* (Participant 02). Furthermore, one participant lamented that *“some pet owners go to pet shops for prescriptions, who [these pet shops] see us as their competitors”* (Participant 04). Many worried that the urbanised city fostering price competition and creating convenience for clients’ vet-hopping, and the current policy may not sufficiently protect animals from seeing non-veterinary trained personnel, which could hinder the animal’s continuity of care from the same veterinarian.

Most participants also reported that the COVID-19 outbreak has had an impact on the veterinary profession. Some claimed that their clinics were busier and had more cases of ingested foreign bodies, suspectedly related to pet owners spending more time working from home with their pets, which led to them having to perform more emergency surgery. Others remarked that their clinics were less busy as clients postponed their pets’ regular check-ups and noticed more cases with serious medical conditions due to delayed check-ups for chronic illnesses. Around half of the participants mentioned that their clinics received COVID-19 precautionary policy-related complaints that restricted the number of clients entering the clinic. One participant recalled, *“Our COVID control measures prohibited clients from visiting their in-hospital pets… They could make video calls, but many clients still wanted to be able to see and touch their pets… I can see why they were upset”* (Participant 13).

Clients’ emotional responses to Hong Kong’s macro-political environment also appeared as a source of stress. A few participants recalled receiving more complaints during the Anti-Extradition Law Amendment Bill Movement, and one claimed that *“the first half of 2020 was terrible. I think the protests and the pandemic worsened the situation. People were way more tense. They had less money and trusted authorities less”* (Participant 18) and believed the upsurge of populism prompted society to question authorities, which was reflected in the profession since they resembled authoritative power.

Our participants identified several contextual and macro-political factors that can influence the stress levels experienced by veterinarians in Hong Kong. Factors unique to Hong Kong included the profession’s relatively short history, the presence of a comprehensive and affordable medical system for humans, and the prevalence of vet hopping behaviour. Societal factors such as white supremacy and age bias also affected veterinarians’ interactions with the AHE community. Furthermore, the COVID-19 pandemic and the political environment also played a role in altering caseloads and provoking clients’ emotional responses. These factors underscore the importance of considering these local challenges when addressing and managing stress experienced by Hong Kong veterinarians.

### 3.6 Theme 4. Veterinarians’ heart: feeling it all

All our participants expressed concerns about fulfilling clients’ expectations and believed miscommunication is the root cause of unmet expectations. Most participants suggested that the most common complaints from clients were related to disagreeable treatment plans, undesired outcomes, or unexpected medical costs. Participants worried that *“if the owner did not like what I do, they’d post it on social media, and of course, I’m always the one at fault”* (Participant 02) and that *“you worry about your reputation, your ability to perform, and your self-esteem”* (Participant 08). Some participants constantly find themselves anxious because *“[when there is a dissatisfied client] it is stressful not knowing what will happen. It could be a complaint, or it could be nothing”* (Participant 14).

Participants felt powerless as they believed they were being unprofessionally judged by the AHE community. One participant believed that *“it [the tasks I do] should really seem to be easy and uneventful if I’m doing my job properly”* (Participant 03). One participant observed that *“so many things now are driven by reviews”* (Participant 09) and worried that clients’ poor online reviews would affect their reputation. Another participant suggested that *“in the old days, the newspaper would, at least, contact us first for our side of the story before publishing anything”* (Participant 12), but the emergence of new media has prioritised rapid reporting and potentially compromised fairness and accuracy. One participant expressed their frustration over negative online comments because *“you cannot defend yourself because your comments can encourage the click rate, and people can get even more excited piling up on you”* (Participant 16). All participants were concerned that negative comments on social media often abandoned medical facts and implied that *“all vets are evil and incapable”* (Participant 18), which can severely damage their reputation.

“If they say, ‘my rabbit died because you were not a good vet’, that’s a bald level of a complaint; but when they say ‘my rabbit died because you did not even want to fix it and did not even care about our animal. You just wanted our money’, that, I think, is the most hurtful” (Participant 18).

Among our participants, all 18 claimed to have known a colleague who had experienced cyberbullying, and 15 claimed to have been a victim of it. Most participants distinguished cyberbullying from negative reviews, as the former involves *“personal attacks on someone whose full name is publicised”* (Participant 08), and the latter is “*someone’s unpleasant experience with the service”* (Participant 10). One participant highlighted that *“in a service industry, you have to be open to the idea that someone’s going to give you a one-star review and say why you are getting one star. That the onus is on me to try to give people a positive experience so that they’ll give me a good rating. But bullying is a different can of worms”* (Participant 09). One participant suggested that social media could “*perk up collective anger where people pile up on us”* (Participant 16). However, one participant pointed out that the root cause of clients’ dissatisfaction is related to unclear communication. *“If you look at the cause of complaints and cyberbullying, it’s the miscommunication… Vets, often, are people who will say ‘Why will not you just accept what I’m saying?’… Clients may not have questioned the vet’s skills, but they were trying to get an understanding of the situation”* (Participant 06).

Furthermore, some participants also felt cynical about the declining standards in the traditional media industry. One participant believed that both traditional and new media compete for swift dissemination of information, which leads to the spread of unverified and false information that has left the profession feeling intimidated and helpless. The participant explained that *“television programmes and digital newspapers report anything eye-catching, even if they contained false information… I regard them as being generous if our names and our clinics’ names were masked”* (Participant 08) and felt frustrated because *“there is no channel to voice our opinion, nobody wants to listen to us”* (Participant 10).

All participants claimed to have known certain clients who had filed tactical complaints, usually through social media, for financial compensation. One participant expressed that *“it makes you think about recording all your conversations and having people sign big, big lists of potential complications… We spend so much time and energy just trying to prevent complaints, where we should be spending it on making pets better”* (Participant 09), to avoid risking their license to practice as a veterinarian. Another participant recalled, *“Clients have said to me, ‘If you do not refund me, I’m going to take you to the vet board’… and you are quite happy to capitulate to get that aggravation taken out of your head”* (Participant 01). Participants admitted to making an effort to prevent complaints.

“I think Hong Kong vets are practising defensively. Say, if you advised your clients about something and, no matter what decisions they made based on your advice, you had better have that down on paper in case they decided to file a complaint against you someday. You’ll need that to defend yourself against VSBHK complaints… But those records do not cease you from cyberbullying” (Participant 14).

Although all participants believed mutual respect between veterinarians and clients is fundamental in veterinarian–client relationships, veterinarians expressed concern about the community’s lack of trust in the profession, and many agreed that *“clients would rather ask a stranger than a vet”* (Participant 04) about their pet’s illnesses and questioned the expensive medical costs. Participants speculated that clients’ unwillingness-to-pay reflected their scepticism about its worthiness, as participants lamented *“Clients do not see our costs. They think every penny goes to profit and our nurses and utilities costs nothing. Even my friends think I’m a scam because we are expensive”* (Participant 11).

“[Once I had a client who brought in an ill pet] I gave them two options: surgery, which had a 95% chance of curing him, or medical treatment, which had a 60% chance… They wrote an online review and said ‘They were trying to steal my money. I chose the cheaper option and my pet still got better’… So, the outcome was good, it was inexpensive, but they still complained” (Participant 18).

Our participants reported that client-related stressors involving unmet expectations and miscommunication can lead to unpredictable actions of dissatisfied clients. This encompasses the potential impact of negative online reviews on their credibility, as they highlighted the role of social media in cyberbullying with personal attacks on veterinarians and the spread of false information, causing anxiety for individuals, clinics, and the veterinary profession as a whole. Participants lamented that the declining standards of traditional media have worsened the situation and are impairing animal welfare, which has led to a decline in trust and respect between veterinarians and clients, resulting in clients seeking advice from strangers rather than veterinarians. Additionally, tactical complaints filed by clients seeking financial compensation further burden the profession, forcing veterinarians into defensive practices to protect their licences from official complaints.

## 4 Discussion

As far as we know, this study represents the first examination of client-related stressors among veterinarians working in East Asia. Through in-depth interviews with 18 Hong Kong-based veterinarians, our research has revealed several key stressors that veterinarians in Hong Kong face. Our findings align with international literature in that veterinarians faced stressors involving client incivility (41), ethical dilemmas (31), clients’ perceived trustworthiness of their veterinarian (42), defensive practising of veterinary medicine (43), and badmouthing through different means, especially on social media (33). Notably, there is little research reporting clients’ vet-hopping behaviour, challenges in multilingual societies, the comparatively short history of veterinary medicine in the city, and psychological projection resulting from socio-political events that are unique to Hong Kong. We believe these unique stressors, along with our prior findings on intra-profession pressure, including business competition and the lack of harmony within the profession (1), necessitate immediate action in mitigating their stress.

It is important to recognise the impact of client-related factors on the stress experienced by veterinarians, as well as the vital role contextual factors play in shaping this phenomenon. Hong Kong’s relatively short history of veterinary medicine, with regulation starting only in 1997 (44), has led to the AHE community’s far-from-reality assumptions about the profession. Clients’ inadequate knowledge of animal welfare, medical risks, and limitations has resulted in unrealistically high expectations, posing a universal stress for veterinarians (7, 45, 46). To address this, it is crucial to promote the visibility of the veterinary profession and facilitate transparent discussions between the AHE community and the professionals (47). We also urge the government to enhance public education on animal welfare by collaborating with professionals in animal care (47, 48), and policymakers should enforce pet owners’ duties of care to prevent animal negligence and cruelty (49). These measures will complement the short history of veterinary medicine in Hong Kong and improve the credibility of the profession for the benefit of veterinarians and animals.

Another significant stressor identified in our research is the presence of negative online reviews on social media platforms, such as Facebook, which serve as an alternative means of lodging complaints that bypasses the lengthy official complaint process. These reviews can damage a veterinarian’s professional reputation and cause considerable stress. Furthermore, the absence of legislation to protect victims of cyberbullying, particularly concerning the involuntary disclosure of personal information, creates a legal grey area surrounding cyberbullying perpetration (50) and adds to the distress. Despite the Hong Kong Government having introduced the anti-doxing law in 2021, their lack of political will to enact the law within professions like veterinarians has resulted in an unresolved matter. We propose that the veterinary profession allocate resources to investigate underlying causes for client complaints and establish a comprehensive system that fosters synchronicity in the provision of veterinary medical services (42) while enhancing training in areas where competencies can be improved.

Furthermore, due to land scarcity and exorbitant rental costs in Hong Kong, veterinary clinics are located in close proximity to each other, leading to the practice of vet-hopping by pet owners. However, this practice disrupts the continuity of veterinary care and results in fragmented medical records, negatively impacting the quality of veterinary medical services (1). To address this, we encourage promoting shared decision-making between veterinarians and clients (10), drawing inspiration from the concept of family medicine in human healthcare (51). Emphasising personalised veterinary care throughout a patient’s life (52) can foster long-term relationships between veterinarians and clients (9), ensuring continuous and high-quality care for animals (9, 10). Additionally, enhancing veterinarians’ interpersonal and communication skills is crucial in establishing veterinarian–client–patient relationships and effectively addressing clients’ concerns (53, 54), which are also skills many young veterinarians lack (54). We recommend incorporating interactional communication in the veterinary curriculum and providing ongoing training for practising veterinarians to broaden their abilities beyond biomedical aspects. Developing well-rounded social–emotional skills through socio-emotional learning can equip individuals to navigate daily challenges, fostering self-awareness, self-regulation, and interpersonal competence.

The primary data for this study was collected during significant political events and public health crises in Hong Kong, which have given rise to populism and anti-intellectualism movements questioning the credibility of authoritative figures (55), including veterinarians (1). These events have had a profound impact on Hong Kong society, leading to heightened stress levels among individuals (1, 56). Given veterinarians’ inherent affinity for animals and the recognised therapeutic effects of the human–animal bond (57), prioritising the exploration of this connection becomes essential in cultivating resilience among veterinarians during times of stress. Further investigation is required to explore the potential role of animals in reducing veterinarians’ stress and building their resilience.

Our findings highlight the importance of considering local challenges when addressing and managing stress experienced by veterinarians in Hong Kong. Addressing these stressors requires a multi-faceted approach involving public education, policy changes, enhanced communication strategies, and improved training for veterinarians. Resilience has become increasingly important in a rapidly changing world. By prioritising these areas, we can contribute to creating a supportive and understanding environment that ultimately benefits the veterinary profession, the AHE community, and the animals they care for.

### 4.1 Limitations

This study has several limitations. Firstly, the qualitative nature of this research aims to describe aspects of participants’ experiences and the depth of the subject, which may not represent the experience of the entire veterinary profession and is not suitable for generalisation. Secondly, the study only included companion animal veterinarians, limiting our understanding of stressors affecting non-companion animal roles, such as laboratory and zoologic veterinarians, who account for less than 10% of all veterinarians practising in Hong Kong (8). Thirdly, although thematic saturation was achieved in this study, the involvement of non-veterinary-trained researchers in the analysis may have introduced subjectivity in determining data saturation. Fourthly, while we acknowledge that selective reporting of stressors originating from the AHE community may raise questions about the range and completeness of our findings, it is important to note that reporting on all identified themes would have diluted the depth of analysis. Given the richness and extensivity of our findings, we believe that selective reporting allows for an in-depth understanding of the specific factors at play. Lastly, although the researchers’ outsider perspectives may limit accessibility to insider information within the veterinary profession, this unique position affords us the advantage of prompting participants to explain and elaborate on various aspects in detail, providing a rich and nuanced understanding of their lived experience as veterinarians.

### 4.2 Implication and future research direction

The findings of the study have several important implications that encompass the well-being of veterinarians and animals (52, 58). By introducing and implementing effective communication strategies within the profession, veterinarians can proactively manage client expectations, improve their communication skills, and prioritise empathetic care to establish trust and foster improved client engagement (9). We also believe improving communication is essential in fostering trust between veterinarians and clients that can contribute to creating a supportive and understanding environment, ultimately benefiting the veterinary profession, the AHE community, and the animals they care for.

Regulating communication quality and meeting clients’ varying expectations present challenges within the veterinary profession. To enhance the legitimacy and respectability of the profession, it is vital to cultivate resilience within the veterinary community and promote transparency. This can be achieved by raising awareness of animal care and distinguishing legitimate information from prevalent misinformation on social media platforms. The veterinary profession combines medical expertise with the business aspect of running clinics. Equipping veterinarians with business knowledge will enable them to navigate smooth business transactions and maximise the potential of their clinics. Further research in this area could explore innovative strategies for business training and development among veterinarians. Furthermore, the socio-political environment and public health crisis can affect societal emotions, including those of veterinarians. Various evidence suggests that human–animal interaction can alleviate psychological tension, improve mental health, and enhance social skills (59). We believe further investigation is warranted in exploring the merits of this bond in mitigating veterinarians’ stress and facilitating discussions on mental health.

## Data availability statement

The datasets for this article are not publicly available due to concerns regarding participant/patient anonymity. Requests to access the datasets should be directed to the corresponding author.

## Ethics statement

The studies involving humans were approved by the Human Research Ethics Committee of the University of Hong Kong (reference number: EA200192). The studies were conducted in accordance with the local legislation and institutional requirements. The participants provided their written informed consent to participate in this study.

## Author contributions

CC conceptualised the original idea, analysed and interpreted the data, and drafted the main manuscript. PW supervised the study design and analysis and critically revised the manuscript. CC and PW agreed on the main focus and ideas of the main manuscript, reviewed the results, and responsible for the content and the writing of the paper. All authors contributed to the article and approved the submitted version.
