# Supplementary material for: Hong Kong veterinarians’ encounters with client-related stress – a qualitative study
Source: Front Vet Sci. 2023 Nov 30;10:1186715. doi: 10.3389/fvets.2023.1186715 (PMC10720360; doi:10.3389/fvets.2023.1186715)
Supplement: Supplementary file 1 [file Data_Sheet_1.pdf]

Supplementary file - interview guide

Opening question:<br>What made you to become a veterinarian?<br>你點解會做獸醫嘅?

1. What are your motivations? What are the rewards?
   你工作嘅動力係啲乜嘢? 你覺得你嘅工作帶畀你啲乜嘢收獲?
   1.1. Who brought you these motivations and rewards? Clients? Patients? Yourself?
   呢啲動力同收獲係邊個帶嚟? 客人? 動物? 自己?

2. What are some day-to-day difficulties or challenges that you face as a vet?
   作為一位獸醫, 你哋日常面對緊啲乜嘢挑戰同困難?
   2.1. Who brought you these difficulties and challenges?
   呢啲挑戰同困難係邊個帶嚟?
   2.2. How do these difficulties and challenges impact your emotions?
   呢啲挑戰同困難對你嘅情緒帶嚟啲乜嘢影響?

3. How do you define a great veterinarian?
   你覺得點樣為之一位傑出嘅獸醫?
   3.1. What does the general public expect from a veterinarian?
   你覺得普羅大眾對獸醫有啲乜嘢期望?
   3.2. What about your clients or pet owners in general?
   咁你嘅客人或者動物嘅主人呢? 佢哋對你哋嘅期望係點?
   3.3. How had these expectations and opinions influenced you?
   想了解吓呢啲期望或者言論對你有啲乜嘢影響?

4. Are you aware of opinions posted by the general public on social media, regarding their comments on the veterinary profession?
   你有冇留意一啲網民會喺社交媒體上面, 討論動物同埋獸醫嘅話題?
   4.1. Who are these people? What are they discussing?
   係乜嘢人討論? 討論啲乜?
   4.2. What's do they expect from veterinarians?
   呢啲網民有冇話對你哋嘅期望係點?
   4.3. How had these expectations and opinions from the Internet influenced you?
   想了解吓呢啲網上嘅期望或者言論, 對你有啲乜嘢影響?

5. Have you ever practiced overseas? Do clients' expectations than those of Hong Kong?
   醫生有冇喺海外執業過? 佢哋嘅客人對獸醫嘅期望, 同香港有冇分別?

6. How do you balance your clients' request and your patients' medical needs? Why?
你會點樣取捨主人嘅要求同動物喺治療上嘅需要? 點解會咁取捨?
    6.1. A client bought in Spot the Dog to Dr. John as Spot wasn't eating and looked sick. Dr. John examined Spot, gave the client a few options, and concluded that a blood test and an x-ray are the best options. The client could not understand why the tests were necessary and accused Dr. John proposed it for his financial benefit. The client was angry and left the clinic. The story was in social media 10 mins after. How do you think Dr. John would feel? What would be your advice to Dr. John?
    一位狗主帶咗佢隻狗, Spot,去睇 Dr. John, 因為 Spot 唔肯食嘢, 同埋佢個樣好唔舒服. Dr. John 了解咗 Spot 嘅症狀之後提供咗幾個唔同嘅方案, 認為驗血同埋照 x 光係最好嘅選擇. 但係狗主唔理解, 認為 Dr. John 為咗呃錢而小事化大, 好嬲咁離開診所. 10 分鐘後, 狗主將依件事放咗去社交媒體. 你覺得 Dr. John 嘅心情係點? 你會畀啲乜嘢建議 Dr. John?
    6.2. Do you often come across with clients not understanding the necessities of medical advice you have given? Why or what didn't they understand?
    會唔會經常遇到啲客唔理解獸醫嘅醫療建議? 你覺得點解佢哋唔理解?
    6.3. Does this bother you? How do you feel about it?
    會唔會因此而感到困擾?
    6.4. Does it affect the vet-client relationship?
    會唔會影響到你同客戶嘅關係?
    6.5. How important is this vet-client relationship?
    你同客戶嘅關係重唔重要?
    6.6. What about the vet-patient relationship?
    咁同動物嘅關係呢? 重唔重要?
    6.7. What can be done, or what should be done, in scenarios like this?
    遇到同類型嘅事件, 你覺得應該要點處理, 或者可以做啲乜?

Now, I would like you learn about your experiences <u>during consultation</u> with your clients and/or patients.
跟住落嚟想醫生你分享一下, 過往診症時嘅一啲經歷

7. What is it like to balance your mental health travelling between the life and death, and the moments of joys and sadness?
你每日嘅診症有機會面對生離死別, 會有開心同唔開心. 你會點樣去調整你起落嘅情緒?
    7.1. Who or what can there be, to make this balancing easier?
    有冇啲乜嘢人或者乜嘢事, 可以令你更容易去調整你呢啲情緒?
    7.2. If you have just had an unpleasant consultation, how do you pick up the pieces and get ready for your next consultation?
    假如你啱啱睇完個症, 令你感覺不愉快, 你會點樣收拾心情, 去繼續接你下一個診症?

7.3. Who or what can there be, so that you can pick up these pieces more quickly?
有冇啲乜嘢人或者乜嘢事, 可以令你更容易好快咁收拾呢啲心情?

8. Can you share with me a good day you have had at work? A memorable one?
醫生可唔可以分享一下以往喺診症時發生過令你印象深刻嘅一件開心嘅事?
   8.1. Who made this day happy? What about the client / patient / others?
   呢件開心嘅事係歸功於邊個? 客人? 動物? 其他?
   8.2. How did the case affect you? Do these motivate you working as a vet?
   呢個經歷對你有啲乜嘢影響? 呢啲係你工作嘅動力嗎?
   8.3. What about your relationship with the client? Was this a good relationship?
   咁你同呢位客戶嘅關係係點? 係一個好嘅關係?
   8.4. What about your patient? Was this a good relationship?
   咁你同動物呢? 係一個好嘅關係?

9. What about a bad day that you remember? What happened?
咁唔開心嘅事呢? 可唔可以分享一下印象深刻嘅事?
   9.1. Who made this day bad? What about the client / patient / others?
   邊個令呢件咁差? 客人? 動物? 其他?
   9.2. How did it you cope with your emotion?
   咁當時你點樣安撫你自己嘅情緒?
   9.3. How did this case affect you as a veterinarian?
   呢個經歷對你有啲乜嘢影響?
   9.4. What about relationship-wise? How did it impact the relationship of these client / patient / colleagues / others?
   如果係關係方面呢? 呢個經歷對你同客人/動物/同事嘅關係有啲乜嘢影響?

Now, I would like you learn about your experiences on the Internet and social media.
跟住落嚟想醫生你分享一下, 過往喺網上同埋社交媒體嘅一啲經歷

10. Have you ever seen yourself being commented on social media? What happened?
你有冇試過畀人點名, 喺社交平台上面討論你? 可唔可以分享發生咗咩事?
   10.1. Who was it? Client? Bystanders?
   係乜嘢人嚟? 客人? 其他?
   10.2. How did you feel?
   你對呢件事有咩感受?
   10.3. Have you seen the client after the incident? Did it feel different?
   呢件事之後你有冇再見過佢? 你哋嘅關係同之前有分別嗎?
   10.4. Did it affect your relationship with the patient?
   同動物嘅關係有冇乜嘢影響?
   10.5. Did it influence those who might saw it? Other clients, colleagues, family and friends?
   有冇影響到, 有機會見到呢個討論嘅人? 其他客人? 同事? 家人? 朋友?
   10.6. How did it you cope with your emotion when you saw it?

你見到網上呢件事嘅時候, 係點樣安撫你自己嘅情緒?

10.7. Did the incident changed how you do things?
呢件事有冇影響你處事嘅方法?

10.8. Would you think this incidence was a cyberbullying? How does it differ from a clients' negative review of your service?
你覺得呢一件事算唔算係網絡欺凌? 你會點樣區分客人對你評論係網絡欺凌, 定係表達緊唔滿意嘅體驗?

11. What if you are to recall a memorable event or something happy happened on social media, can you share that experience with me?
如果要醫生你分享一下 以往喺網上媒體發生過 令你印象深刻 嘅一件開心嘅事，你會諗起乜嘢事呢?

11.1. Who made this happy? What about the client / patient / others?
呢件開心嘅事係歸功於邊個? 客人? 動物? 其他?

11.2. How did it make you feel? Does it feel like your work has been recognised?
你對呢件事有啲乜嘢感受? 有被認同嘅感覺?

11.3. What about your relationship with the client? Was this a good relationship?
咁你同呢位客戶嘅關係係點? 係一個好嘅關係?

11.4. What about your patient? Was this a good relationship?
咁你同動物呢? 係一個好嘅關係?

12. Overall, how well do you think veterinarians are coping with mental distress?
整體嚟講, 你覺得獸醫業界喺應對情緒壓力方面, 應對得理唔理想?

12.1. Do you think the extends of mental distress and the ability to cope varies from clinic-to-clinic?
你覺得唔同嘅診嘅情緒壓力, 或者對於呢啲壓力嘅應對, 會唔會有所唔同?

12.2. Were you aware of these mental distresses before you entered the profession?
你執業之前係咪已經知道會遇到呢啲情緒壓力?

12.3. How did you learn about self-care? In vet school? Did you think it was helpful?
你係點樣學習點樣安撫自己嘅情緒? Vet school? 你覺得有冇用?

12.4. What about conflict resolution? How was it learnt?
咁處理唔同嘅觀點或拗撬? 你係喺邊度學?

13. When it comes to distress, how do you relieve your emotions?
你平時會用啲咩方法去舒緩呢啲情緒?

13.1. What if these didn't work? Will you seek for other support? What sort of support would you seek for?
如果用完呢啲方法都舒緩唔到, 你會唔會尋求其他形式去協助處理你嘅情緒?

13.2. Have seeking professional mental health advice ever crossed your mind? Why?
有冇諗過可以尋求專業人仕嘅協助? 點解呢?

14. Typically, do your clients' beliefs or attitudes influence your mental well-being? How?
喺日常診症, 客人嘅態度同埋睇法對你嘅情緒有冇影響? 點影響呢?

14.1. What about your patients? How do they influence your mental well-being?

咁動物呢? 佢哋對你嘅情緒健康有啲乜嘢影響?

14.2. What about the health status of the patient? How does it affect your emotions?
咁隻動物嘅健康程度, 對你嘅情緒影響呢?

15. What do you think of your own mental health? How important is it to you?
你覺得你自己嘅情緒健康係點? 有幾重視你嘅情緒健康?

15.1. Are you aware of any mental health support services available in Hong Kong, specifically for veterinarians? Is there a necessity?
以你所知, 香港有冇一啲提供畀獸醫業界嘅情緒支援服務? 有冇咁嘅需要呢?

15.2. What types of mental health support, do you think, are useful for vets?
你認為乜嘢類型嘅支援服務有助於醫獸業界維持佢哋嘅情緒健康?

15.3. In some countries, they are offering counselling services and mental health support specifically for veterinarians. Do you think it will be useful if we have it in Hong Kong? Why?
依家有一啲國家提供緊一啲專門為獸醫而設嘅情緒輔導同埋支援服務, 你覺得如果香港都有呢類型嘅服務, 會唔會有用? 點解?

Ending Question:
What do you think are the most realistic ways to maintain veterinarians' mental well-being?
你覺得咩方法係最實際最有效去維持獸醫嘅情緒健康?
